# Immunomodulatory Properties of a γ-Aminobutyric Acid-Enriched Strawberry Juice Produced by *Levilactobacillus brevis* CRL 2013

**DOI:** 10.3389/fmicb.2020.610016

**Published:** 2020-12-17

**Authors:** Pablo G. Cataldo, Julio Villena, Mariano Elean, Graciela Savoy de Giori, Lucila Saavedra, Elvira M. Hebert

**Affiliations:** Centro de Referencia para Lactobacilos (CERELA-CONICET), San Miguel de Tucumán, Argentina

**Keywords:** GABA, lactic acid bacteria, *Levilactobacillus brevis*, strawberry fermented juice, anti-inflammatory properties, TLR4 activation

## Abstract

Gamma-aminobutyric acid (GABA) plays a key role in mammals as the major inhibitory neurotransmitter of the central nervous system. Although GABA may not be able to cross the human blood-brain barrier, it was approved as a food ingredient because of its benefits to the host after oral administration including anti-hypertensive, anti-depressant and anti-inflammatory activities. Considering the current trend toward the development of new functional and natural products and that microbial fermentation is one of the most promising methods to produce this non-protein amino acid, the *in situ* production of GABA through fermentation of strawberry and blueberry juices by the efficient GABA producer strain, *Levilactobacillus brevis* (formerly known as *Lactobacillus brevis*) CRL 2013, was evaluated. A high GABA production (262 mM GABA) was obtained after fermenting strawberry juice supplemented with yeast extract for 168 h, being GABA yield significantly higher in strawberry juices than in the blueberry ones. Thus, GABA-enriched fermented strawberry juice (FSJ) was selected to carry out *in vivo* and *in vitro* studies. The *in vitro* functional analysis of the GABA-enriched FSJ demonstrated its ability to significantly decrease the expression of *cox-2* gene in LPS stimulated RAW 264.7 macrophages. In addition, *in vivo* studies in mice demonstrated that both, *L. brevis* CRL 2013 and the GABA-enriched FSJ were capable of reducing the levels of peritoneal, intestinal and serum TNF-α, IL-6, and CXCL1, and increasing IL-10 and IFN-γ in mice exposed to an intraperitoneal challenge of LPS. Of note, the GABA-enriched FSJ was more efficient than the CRL 2013 strain to reduce the pro-inflammatory factors and enhance IL-10 production. These results indicated that the CRL 2013 strain exerts anti-inflammatory effects in the context of LPS stimulation and that this effect is potentiated by fermentation. Our results support the potential use of *L. brevis* CRL 2013 as an immunomodulatory starter culture and strawberry juice as a remarkable vegetable matrix for the manufacture of GABA-enriched fermented functional foods capable of differentially modulating the inflammatory response triggered by TLR4 activation.

## Introduction

Gamma-aminobutyric acid (GABA) is an ubiquitous non-protein amino acid widely distributed among microorganisms, plants and animals, having diverse physiological functions and great potential health benefits (Ramos-Ruiz et al., 2018). GABA exerts some positive effects on mammalian physiology; such as hypotensive, relaxation, antidiabetic and immunity enhancement effects (Adeghate and Ponery, 2002; Abdou et al., 2006; Yang et al., 2012). Due to its relevance and health benefits, GABA is becoming recognized as an essential nutrient for a healthy and balanced diet. GABA has been authenticated as new resource food by China Food and Drug Administration in 2009 and it is listed in the Chinese Pharmacopeia (Ramos-Ruiz et al., 2018). Additionally, a healthy diet following the WHO food-based dietary guidelines (FBDG) and/or the Healthy Eating Plate (Harvard) will provide a considerable amount of GABA as a natural nutrient. Nevertheless, GABA content in natural animal- and plant-based food products is low (Quílez and Diana, 2017). Therefore, efforts are being devoted to the development of new technological processes for GABA enrichment in traditional foods.

Lactic acid bacteria (LAB) are microorganisms that inhabit nutrient-rich environments associated with food, seeds, plants, animals, and humans. Due to their Qualified Presumption of Safety (QPS) status and their use in food, biotechnology, and therapeutic applications, LAB are highly industrially relevant microorganisms and represent a multi-billion Euros business worldwide (Johansen, 2017). Thus, LAB strains, mainly belonging to *Levilactobacillus brevis* (formerly known as *Lactobacillus brevis*) species, constitute the most competitive and technologically relevant group of microorganisms used to synthesize GABA since they are able to produce high levels of this compound within a variety of food matrices (Park and Oh, 2007; Kim et al., 2009; Wu et al., 2015; Li et al., 2016; Villegas et al., 2016; Bao et al., 2020; Cui et al., 2020). In this regard, we have recently demonstrated that *L. brevis* CRL 2013 is an efficient microorganism for the conversion of monosodium glutamate (MSG) to GABA in hexose-supplemented complex media with conversion ratios about 99% (Cataldo et al., 2020). This GABA yield was one of the highest values observed among lactobacilli grown in batch culture (Villegas et al., 2016; Cataldo et al., 2020; Cui et al., 2020). Then, our results support the potential use of *L. brevis* CRL 2013 as a starter culture for the manufacture of GABA-enriched functional foods.

In dietary guidelines worldwide, an increased consumption of fruits and vegetables is recommended. The intake of the so-called “superfruits” like berries that are rich in nutrients, phytochemicals and constitute important dietary reservoirs of bioactive compounds can prevent various diseases and disorders (Nile and Park, 2014). Thus, berries have potent antioxidant, anticancer, antimutagenic, antimicrobial, anti-inflammatory, and anti-neurodegenerative properties, both *in vitro* and *in vivo* (Nile and Park, 2014). Among berries, blueberry (*Vaccinium corymbosum*) and strawberry (*Fragaria x ananassa*) are widely cultivated in Argentina, which is the second largest strawberry producer in The Common Market of the Southern Cone (MERCOSUR), being Canada, United States, and the European Union the main export destinations of Argentinian strawberries. These fruits are not only available fresh but also generally consumed frozen and processed into juices, yogurts, beverages, jams, and jellies. In general, berries have a low GABA content, ranging from 0.016 mg/g for strawberries to 0.079 mg/g for blueberries (Ramos-Ruiz et al., 2018). Fortifying beverages with GABA is being intensively studied due to its potential health benefits (Kim et al., 2009; Quílez and Diana, 2017). However, artificially produced GABA cannot be added to food manufacture since GABA is not a legal additive in several countries; including countries of the European Union and Argentina [Efsa Panel on Dietetic Products, and Nutrition, and Allergies [NDA], 2009; Kim et al., 2009]. Then, the use of efficient GABA producer starter cultures such as *L. brevis* CRL 2013 to increase GABA concentrations in berry-juices would be an alternative to bioenrich these beverages with this bioactive compound. Therefore, the aims of this work were: (a) to obtain a GABA-enriched berry juice fermented by the high GABA producer *L. brevis* CRL 2013, combining the health benefits of both, GABA and berries; and (b) to evaluate the potential anti- inflammatory properties of this GABA-enriched juice by using *in vitro* and *in vivo* approaches in the context of Toll-like receptor (TLR)-4 mediated inflammation.

## Materials and Methods

### Microorganism and Growth Conditions

*Levilactobacillus brevis* CRL 2013 was isolated from Andean Real Hornillos quinoa sourdough (Cataldo et al., 2020) and belongs to the CERELA culture collection (CERELA-CONICET, Argentina). The strain was routinely propagated and cultivated in a modified MRS broth (pH 6.5) containing 1% glucose and 1% fructose instead of 2% glucose. When assessing GABA production, cells were statically grown in capped test tubes containing 267 mM of monosodium glutamate (MSG) at 30°C. For the juice fermentation assays, eighteen-hour cultures were harvested by centrifugation (9,000 × *g* for 10 min), washed twice with sterile 0.8% (w/v) NaCl and used as inoculums to reach an initial optical density at 600 nm (OD_600_) of about 0.1 (approximately 5 × 10^7^ CFU/ml).

### Determination of GABA Concentration

Gamma-aminobutyric acid was tittered using a modified version of the GABase method previously described by Tsukatani et al. (2005). Briefly, 86 mM Tris–HCl buffer (pH 9), 5 mM α-ketoglutarate, 3.3 mM 2-mercaptoethanol, 1.2 mM NADP^+^, and 0.03 U of GABase were added to each well of a 96-well microtiter plate. The mixture was warmed at 25°C, and then the standard or sample solution (fermented juice supernatants) was added (Cataldo et al., 2020). The NADPH formation was measured at 340 nm every 1 min for 10 min at 25°C in a Biotek Synergy HT microplate reader (Winooski, VT, United States). GABA concentration in each sample was calculated from the calibration curve of the standard solutions (0.10, 0.25, 0.50, and 1 mM GABA).

### Development of GABA-Enriched Fermented Juices

Blueberries (*Vaccinium corymbosum*) and strawberries (*Fragaria x ananassa*) harvested in 2018 were purchased from production fields in Lules (Tucumán, Argentina). For the preparation of blueberry (BJ) and strawberry (SJ) juices, frozen fruits were thawed at room temperature for 4 h and then crushed on a Russell Hobbs JM550SRH Juice extractor (China). Juices were then centrifuged three times at 14,000 × *g* for 10 min to get rid of the remaining pulp and then sterilized at 115°C for 15 min. When needed, pulp-free juices were supplemented with 267 mM MSG, 1% (w/v) yeast extract (YE) or tryptein (T). The initial pH was either adjusted to approximately 6.5 with 0.1 M NaHCO_3_ or left unmodified. All juices were inoculated with *L. brevis* CRL 2013 to an initial cell density of 5 × 10^7^ CFU/ml. Cell growth (OD_600_), pH and GABA production were determined at different time intervals for 7 days. To determine cell growth, bacterial culture was washed twice with phosphate buffered solution (PBS) and resuspended to the original volume using the same solution. Non-inoculated berry juices processed in the same way as the sample were used as controls. Juices fermentations were followed up until the conversion rate from MSG to GABA reached its maximum values for this strain (around 98%).

### Cell Culture

Mouse RAW 264.7 macrophages were obtained from IMBICE, CONICET (Argentina). Cells were routinely kept in a RPMI Medium (Genbiotech, Argentina), supplemented with 10% (w/v) fetal bovine serum (FBS, NATOCOR, Argentina), penicillin G (100 U/ml, Gibco, ThermoFisher, Argentina), streptomycin (100 μg/ml, Gibco) and amphotericin B (25 μg/ml, Gibco) at 37°C in a humidified 5% CO_2_-95% air incubator (standard conditions).

### MTT Cell Viability Assay

The influence of GABA-enriched berry juices on cell proliferation was analyzed by the 3-(4, 5-dimethylthiazol-2-yl)-2, 5-diphenyltetrazolium bromide (MTT) assay (Marcial et al., 2014). Briefly, RAW 264.7 cells were seeded at 5.0 × 10^4^ cells per well with RPMI containing 10% FBS in 96-well plates and cultured for 24–48 h at 37°C in a humidified environment until 85% of confluence was reached. Then, the medium was removed, cells were washed twice with PBS and adherent cells were incubated at 37°C for 24 h in the presence of GABA (0.1 and 1.0 mM) (Sigma-Aldrich Co., MO, United States), filtrated dilutions of fermented strawberry juice (FSJ) supernatants to obtain a final GABA concentration of 1 mM or the same dilution of the non-fermented strawberry juice (NFSJ). After incubation, the medium was discarded, cells were washed with PBS and 50 μl of MTT solution (2.6 mg/ml in PBS) were added in each well and incubated for 4 h at 37°C in a 5% CO_2_ incubator. Living cells convert MTT into a purple colored formazan product. MTT was removed from the plates and 50 μl DMSO/well were added to dissolve the formazan crystals. After 5 min the absorbance at 570 nm, which is directly proportional to the cellular metabolism, was measured in a Biotek Synergy HT microplate reader (Winooski, VT, United States) and cell viability was estimated as the percentage absorbance of sample relative to the positive control.

### RT-qPCR

RAW 264.7 cells were inoculated in 6-well plates (0.5 × 10^6^ cells/ml) and incubated as described above for 24–48 h. At 85% confluence, the medium was replaced with fresh media containing GABA (0.1 or 1 mM), filtrated dilutions of FSJ supernatants to obtain a final GABA concentration of 1 mM or the same dilution of the NFSJ, and cells were pre-incubated for 1 h at 37°C. Then, cells were treated with 1 μg/ml of lipopolysaccharide (LPS) for 4 h to induce the inflammatory state. Adherent cells were harvested after the LPS stimulation and total RNA was isolated using the TRIzol reagent following the manufacturer’s instructions (Life Technologies, Buenos Aires, Argentina). Briefly, cells were lysed in 400 μl of TRIzol; and 200 μl of chloroform were added to each tube. Finally, the suspensions were centrifuged for 15 min at 1,2000 × *g* and 4°C. RNA was isolated from the upper hydrophilic phase by adding 100 μl of isopropyl alcohol and allowing it to precipitate for 2 h at −20°C. Samples were centrifuged again, the supernatants were discarded and pellets were washed twice with 70% cold ethanol, resuspended in 15 μl of RNase-free water and stored at −80°C. Total RNA was quantified using the Qubit RNA HS assay kit (Life technologies). The reverse transcription was carried out employing the SuperScript III First Strand Kit (Life technologies) following the supplier’s instructions.

The specific oligonucleotides sequences for cyclooxygenase (*cox-2*) gene and β-actin used in this study were previously described by Chang et al. (2016). Real Time qPCR was performed on an iQ5 Real-Time PCR Detection System (BioRad) with the IQTM SYBR^®^supermix (Bio-Rad) in 96-well plates. PCR was performed with 1 μl of cDNA (100 ng) or water in the non-template controls, 4 μl of primer mix (0.3 μM of each primer), 5 μl of RNase-free water and 10 μl of IQTM SYBR^®^supermix as described by Brown et al. (2017). The PCR cycles consisted in: 95°C for 4 min, 40 cycles of 95°C for 15 seg, 52°C for 30 seg, 72°C for 30 seg and 95°C for one-minute. A non-template control was included within each PCR reaction. Amplification efficiencies were validated and normalized against the β-actin gene. A melting curve analysis was performed immediately at the end of each experiment at a linear temperature transition rate of 0.1°C/s from 55 to 95°C to determine the specificity of the amplification. The relative mRNA expression (as fold change) was determined using β-actin as normalizing housekeeping gene by the 2^–ΔΔCT^ or Livak method (Livak and Schmittgen, 2001).

### Animals and Feeding Procedures

Male 4-week-old Balb/c mice were obtained from the closed colony kept at CERELA (Tucuman, Argentina). They were housed in plastic cages with controlled room temperature (22 ± 2°C temperature, 55 ± 2% humidity) and were fed *ad libitum* conventional balanced diet. This study was carried out in strict accordance with the recommendations of the Guide for the Care and Use of Laboratory Animals of the Guidelines for Animal Experimentation of CERELA. The CERELA Institutional Animal Care and Use Committee prospectively approved this research under the protocol BIOT-CRL-17.

Mice were housed individually during the experiments and the assays for each parameter studied were performed in five mice per group. *L. brevis* CRL 2013 was administered to mice for 3 consecutive days at a dose of 2 × 10^8^ cells/mouse/day in the drinking water (4 ml per mice per day). Different groups of mice were also treated with GABA-enriched fermented strawberry juice (FSJ) containing ∼140 mM GABA or diluted (1:2) GABA-enriched FSJ containing ∼70 mM GABA [FSJ + CRL2013 and FSJ + CRL2013 (d), respectively] during 3 consecutive days *ad libitum*. The GABA-enriched fermented strawberry juice (FSJ + CRL2013) contains about 2.7 × 10^7^ CFU/ml. Groups of mice treated with non-fermented strawberry juice (NFSJ) or NFSJ supplemented with yeast extract (NFSJ + YE) were used as controls. One day after the end of the treatments, mice were challenged with LPS to induce inflammation as described previously (Garcia-Castillo et al., 2019). Mice received 8 mg/kg of LPS from *Escherichia coli* O55:B5 by intraperitoneal injection.

### Cytokine Concentrations

The concentration of cytokines was determined in peritoneal fluid, blood and intestinal samples. Peritoneal fluid was collected from mice as described previously (Garcia-Castillo et al., 2019). Blood samples were obtained through cardiac puncture at the end of each treatment and collected in heparinized tubes. Intestinal fluid samples were obtained by separating the small intestine and flushing it with 5 ml of PBS. The fluid was centrifuged (10,000 × *g*, 4°C for 10 min) to separate particulate material. The supernatant was kept frozen until use. Blood and tissue samples were obtained from mice after the intraperitoneal injection of ketamine (80 mg/kg) and xylazine (10 mg/kg) according to the recommendations of the CERELA Institutional Animal Care and Use Committee.

Tumor necrosis factor α (TNF-α), interferon γ (IFN-γ), interleukin (IL)-10, IL-6, and the IL-8 mouse homolog chemokine KC or chemokine (C-X-C motif) ligand 1 (CXCL1) concentrations were measured with commercially available enzyme-linked immunosorbent assay (ELISA) kits following the manufacturer’s recommendations (R&D Systems, MN, United States). TNF-α (Mouse TNF-α ELISA Kit, sensitivity: 1.5 pg/ml), IFN-γ (Mouse IFN-gamma Quantikine ELISA Kit, sensitivity: 2 pg/ml), IL-6 (Mouse IL-6 Quantikine ELISA Kit, sensitivity: 1.8 pg/ml), IL-10 (Mouse IL-10 Quantikine ELISA Kit, sensitivity: 5.2 pg/ml), and CXC1 (Mouse CXCL1/KC DuoSet ELISA, sensitivity 2.3 pg/ml) kits were used.

### Intestinal Tissue Injury

Intestinal tissue injury was evaluated as described previously (Elean et al., 2020). Briefly, small intestine samples from all experimental groups were excised and washed out with PBS. Then, tissues were immersed in 4% (v/v) formalin saline solution. Once fixed, samples were dehydrated and embedded in Histowax (Leica Microsystems Nussloch GmbH, Nussloch, Germany) at 56°C. Finally, intestines were cut into 4 μm serial sections and stained with hematoxylin-eosin for light microscopy examination. All slides were coded and evaluated blindly. A semiquantitative scoring index was used to evaluate alterations in the intestine. The presence/absence and intensity of edema, epithelial injury, degranulation of Paneth cells and inflammation were considered. Each parameter was rated on a point damage scale from 1 to 4 (1, absence; 2, slight; 3, moderate; 4, severe alteration) and the final score results were expressed as the sum of the individual scores given to each parameter (Torres et al., 2017).

### Statistical Analyses

Statistical analyses were performed with the software package Minitab 17 (Minitab Inc.) using ANOVA general linear models followed by Tukey’s *post hoc* test where *p* < 0.05 was considered significant. Unless otherwise specified, all reported values were the means of three independent trials ± standard deviation. No significant differences were observed between individual replicates.

## Results

### Formulation of GABA-Enriched Fermented Berry Juices

**FIGURE 1:**
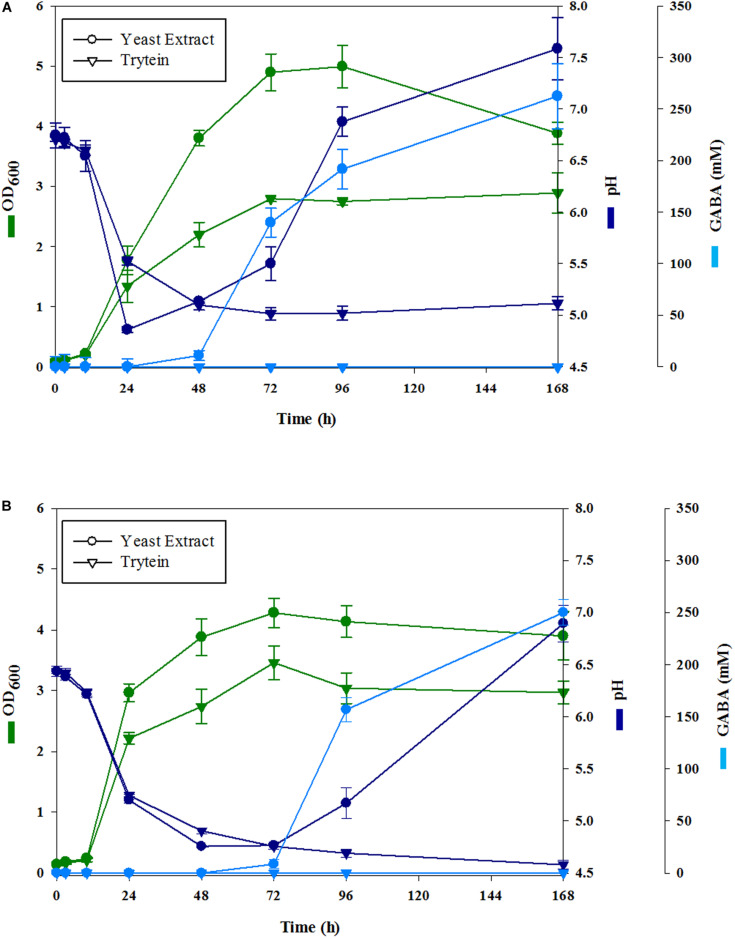
Cell growth (green lines), pH (blue lines), and GABA production (cyan lines) by *L. brevis* CRL 2013 in MSG strawberry **(A)** and blueberry **(B)** juices supplemented with yeast extract (•) or tryptein (▼) at an initial pH of 6.5. Cell growth is expressed as ln x/x0, where x0 is initial biomass, and x is biomass at the indicated time. All values are means ± standard deviations from at least three separate experiments.

In order to develop GABA-enriched fermented berry juices, firstly, we studied the growth of *L. brevis* CRL 2013 in blueberry and strawberry pulp-free juices supplemented with 267 mM MSG. As control, the strain was grown in the same juices without extra supplementation. GABA production was not observed in the fermented berry juices supplemented only with MSG (data not shown). Thus, growth parameters and GABA production were evaluated in the pulp-free juices supplemented with MSG and YE or tryptein. Initial pH was either adjusted to *ca*. 6.5 using 0.1 M NaHCO_3_ or left unmodified and the fermentation was monitored for 7 days at 30°C. No significant differences in cell density and pH time course were observed between juices with adjusted and unadjusted initial pH (data not shown). In both YE and tryptein-supplemented media, *L. brevis* CRL 2013 was able to reach a sustained growth (Figure 1). YE-supplemented juices presented higher cell densities than those observed for the tryptein-supplemented ones (Figure 1). In the first 24 h of fermentation, the cell growth was accompanied by a decrease in the pH of the media independently of the initial pH. Thereby, pH values for strawberry and blueberry juices sharply decreased during the first 24 h and 48 h, reaching values of ∼4.86 and ∼4.75 for the YE supplemented strawberry (Figure 1A) and blueberry juices (Figure 1B), respectively.

Although juices supplemented with tryptein allowed a sustained growth of *L. brevis* CRL 2013, no increase in the pH of the medium was observed and GABA was not detected in the culture supernatants. Contrariwise, in strawberry juice supplemented with YE (Figure 1A), GABA synthesis began after 24 h of fermentation whereas in blueberry juice (Figure 1B), GABA became detectable only after 48 h. In both cases, GABA production was accompanied by a concomitant increase of the pH of the extracellular milieu (Figure 1). In YE-supplemented juices GABA production progressed up to 168 h. Thus, the highest productivity (262 mM GABA) was obtained after fermenting strawberry juice supplemented with YE for 168 h, being GABA yield significantly higher in strawberry juices than in the blueberry ones. No interference by other compounds was found during GABA determinations and GABA was not detected in any of the intact juices without MSG supplementation. Based on these results, we selected the GABA-enriched fermented strawberry juice (FSJ) for 72 h as a model for further functional studies. This juice contains about 2.7 × 10^7^ CFU/ml and a GABA concentration of around 140 mM, which would be compatible with the doses reported by Taranukhin et al. (2017) to carry out functional assays.

### Anti-inflammatory Effect of GABA-Enriched FSJ on Mouse Macrophages

We aimed to evaluate *in vitro* whether the GABA-enriched FSJ was able to modulate the response of macrophages to TRL4 activation. For this purpose, we first studied whether the GABA-enriched FSJ was able to exert any detrimental effect on RAW 264.7 macrophages by evaluating their viability (Supplementary Figure 1). Then, mouse macrophages were stimulated with GABA-enriched FSJ that was diluted until reaching a GABA concentration equal to 1 mM. This final amount of GABA was selected considering that this concentration was the optimal to modulate the expression of inflammatory factors in RAW 264.7 cells (Han et al., 2007). In addition, NFSJ as well as stimulations with 0.1 and 1.0 mM GABA were used for comparisons. All the treatments yielded survival percentages around 82% (Supplementary Figure 1), which allowed the use of these samples for pro-inflammatory marker expression studies. No significant differences were observed in the viability of macrophages after their treatment with both fermented and non-fermented juices or GABA alone.

**FIGURE 2:**
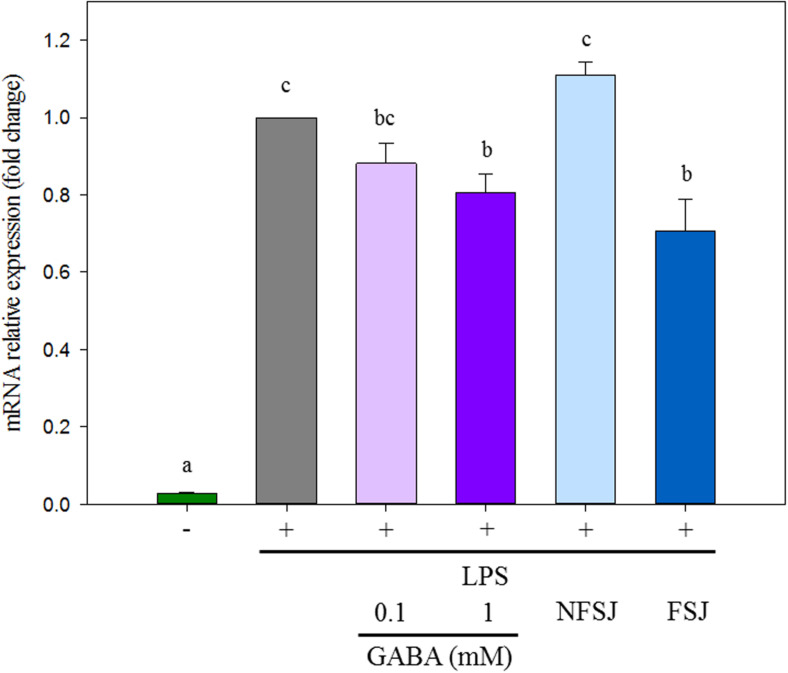
Effects of GABA, non-fermented strawberry juice (NFSJ) and GABA-enriched fermented strawberry juice (FSJ) on *cox-2* expression in RAW 264.7 cells stimulated by LPS. After treatment with the indicated compounds for 1 h, cells were stimulated for 4 h with LPS (1 μg/ml). Total RNA was isolated, and then RT-qPCR was used to measure the mRNA levels of *cox-2* with *β-actin* expression as an internal control. Data represent means ± standard error of three independent experiments. Means for each bar without a common letter differ significantly. Significance with Tukey’s HSD *post hoc* test following a one-way ANOVA is indicated as *p* < 0.05.

The potential anti-inflammatory properties of the GABA-enriched FSJ were assessed through the evaluation of the relative expression of a key factor that is up-regulated in response of TLR4 activation: COX-2 (Lee et al., 2003). As expected, control macrophages stimulated with LPS significantly increased the expression levels of *cox-2* (Figure 2). The treatment of macrophages with 0.1 mM GABA did not induce remarkable effects in the response of these cells to the activation of TLR4, however, when GABA was used at a concentration of 1 mM, a significant reduction of *cox-2* transcripts was observed compared to LPS-challenged control cells (Figure 2). Similarly, mouse macrophages treated with GABA-enriched FSJ had significantly lower levels of *cox-2* than LPS-challenged controls. Moreover, the expression of *cox-2* in macrophages treated with GABA-enriched FSJ was not different from that found in cells stimulated with 1 mM GABA. No significant differences in the expression of *cox-2* were observed when control macrophages and cells treated with NFSJ were compared (Figure 2). These results indicated that the GABA-enriched FSJ obtained after fermentation with the *L. brevis* CRL 2013 strain has the potential to beneficially modulate the TLR4-mediated inflammation.

### Effect of GABA-Enriched FSJ in Mice Cytokine’s Profile

**FIGURE 3:**
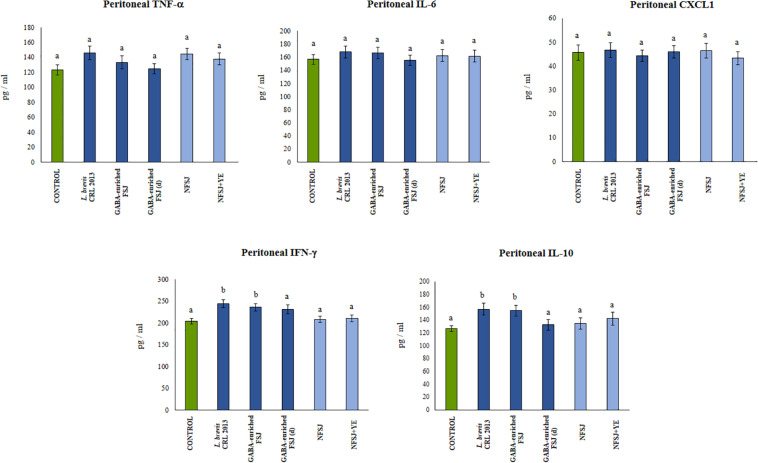
Effect of *L. brevis* CRL 2013, GABA-enriched strawberry fermented juice (FSJ) and diluted (d) GABA-enriched FSJ on peritoneal cytokines and chemokines in Balb/c mice. Animals treated with NFSJ or NFSJ supplemented with YE were used for comparisons. Results are expressed as mean ± SD. Means for each bar without a common letter differ significantly. Significance with Tukey’s HSD *post hoc* test following a one-way ANOVA is indicated as *p* < 0.05.

**FIGURE 4:**
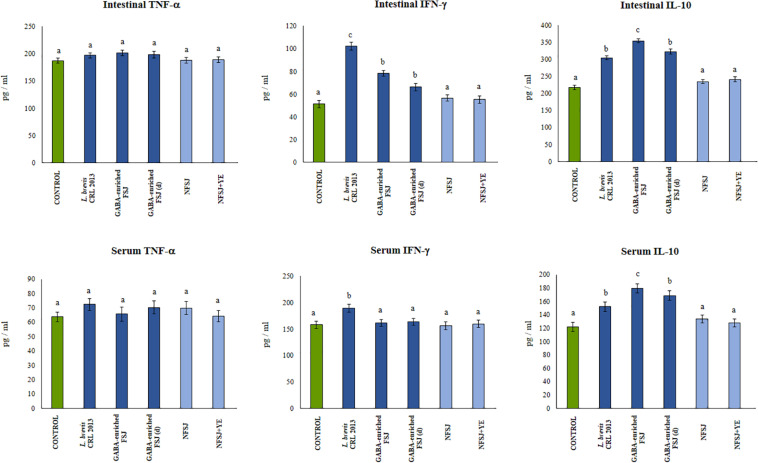
Effect of *L. brevis* CRL 2013, GABA-enriched strawberry fermented juice (FSJ) and diluted (d) GABA-enriched FSJ on intestinal and serum cytokines and chemokines in mice. Animals treated with NFSJ or NFSJ supplemented with YE were used for comparisons. Results are expressed as mean ± SD. Means for each bar without a common letter differ significantly. Significance with Tukey’s HSD *post hoc* test following a one-way ANOVA is indicated as *p* < 0.05.

We next aimed to evaluate *in vivo* the effect of the GABA-enriched FSJ. For this purpose, mice were treated with the GABA-enriched FSJ or the diluted GABA-enriched FSJ (1:2) during 3 days. In addition, to assess the potential intrinsic immunomodulatory effect of the bacterial strain, a group of animals treated only with *L. brevis* CRL 2013 was included. Animals treated with NFSJ or NFSJ supplemented with YE were also used for comparisons. When the levels of the pro-inflammatory factors TNF-α, IL-6 and CXCL1 were evaluated in the peritoneal cavity after treatments with GABA-enriched FSJ, diluted GABA-enriched FSJ or *L. brevis* CRL 2013 no significant differences were found between these groups or the untreated control mice (Figure 3). Similarly, no differences in the levels of intestinal and serum TNF-α were detected when GABA-enriched FSJ- or CRL 2013-treated mice were compared to controls (Figure 4). Of note, mice receiving GABA-enriched FSJ or *L. brevis* CRL 2013 had higher levels of peritoneal IFN-γ and IL-10 than control animals, an effect that was not observed in mice receiving the diluted GABA-enriched FSJ (Figure 3). In addition, mice treated with GABA-enriched FSJ or *L. brevis* CRL 2013 had higher levels of intestinal IFN-γ and IL-10 than control animals (Figure 4). The CRL 2013 strain was more efficient than the GABA-enriched FSJ and the diluted FSJ to increase the intestinal IFN-γ. Interestingly, the GABA-enriched FSJ was more efficient than *L. brevis* CRL 2013 and the diluted FSJ to increase the intestinal IL-10. Only *L. brevis* CRL 2013 was able to increase the levels of serum IFN-γ when compared to controls (Figure 4). In addition, although the treatments with CRL 2013 and GABA containing juices increased the levels of serum IL-10, the treatment with non-diluted GABA-enriched FSJ was more efficient to induce this effect (Figure 4). Animals treated with NFSJ or NFSJ supplemented with YE had values of peritoneal (Figure 3), intestinal and serum cytokines (Figure 4) that were not different from untreated control mice.

### Anti-inflammatory Effect of GABA-Enriched FSJ in Mice

**FIGURE 5:**
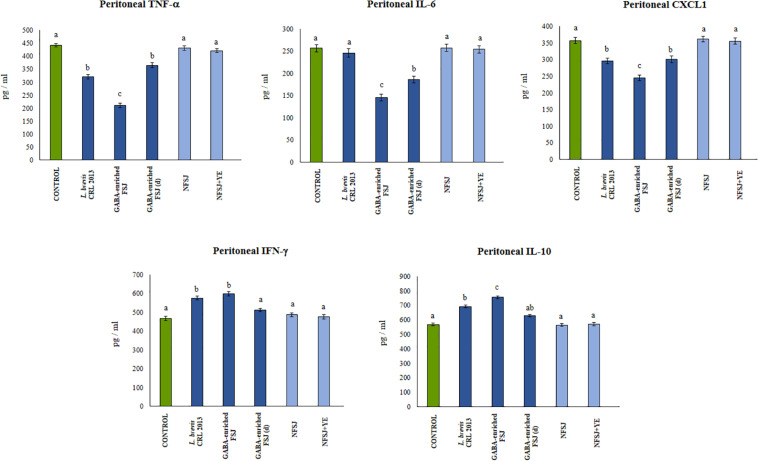
Effect of *L. brevis* CRL 2013, GABA-enriched strawberry fermented juice (FSJ) and diluted (d) GABA-enriched FSJ on peritoneal cytokines and chemokines in mice challenged with LPS. Animals treated with NFSJ or NFSJ supplemented with YE were used for comparisons. Results are expressed as mean ± SD. Means for each bar without a common letter differ significantly. Significance with Tukey’s HSD *post hoc* test following a one-way ANOVA is indicated as *p* < 0.05.

Finally, we aimed to confirm our *in vitro* findings by evaluating *in vivo* the anti-inflammatory effects of GABA-enriched FSJ in the context of TRL4 activation. Then, animals received the five treatments described before and on the day 4 they were challenged with an intraperitoneal injection of LPS. The activation of TLR4 significantly increased levels of the pro-inflammatory factors TNF-α, IL-6 and CXCL1 as well as IFN-γ and IL-10 in the peritoneal cavity (Figure 5). Mice receiving the GABA-enriched FSJ showed the most remarkable differences in the peritoneal cytokines ’profile. This group of mice had significantly lower levels of TNF-α, IL-6 and CXCL1 and higher concentrations of IFN-γ and IL-10 when compared to LPS-challenged controls. The treatment with diluted GABA-enriched FSJ was also capable of reducing the levels of inflammatory factors and slightly increases IL-10; however, it did not achieve the effect induced by the non-diluted GABA-enriched FSJ (Figure 5). Of note, mice treated with *L. brevis* CRL 2013 had reduced levels of TNF-α and CXCL1 that were similar to those found in the diluted GABA-enriched FSJ group. The CRL 2013 strain was not able to modify the levels of IL-6 when compared to controls. In addition, mice treated with *L. brevis* CRL 2013 had increased levels peritoneal IFN-γ and IL-10. Whereas the levels of IFN-γ were similar to those found in the GABA-enriched FSJ group, the levels of IL-10 were significantly lower (Figure 5).

**FIGURE 6:**
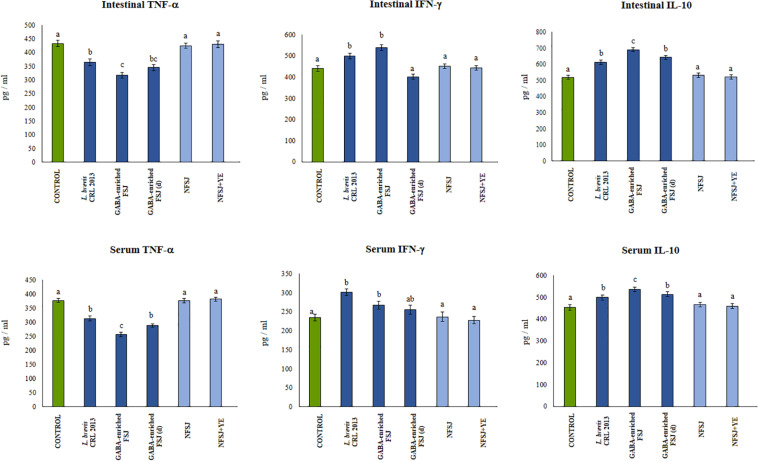
Effect of *L. brevis* CRL 2013, GABA-enriched strawberry fermented juice (FSJ) and diluted (d) GABA-enriched FSJ on intestinal and serum cytokines and chemokines in mice challenged with LPS. Animals treated with NFSJ or NFSJ supplemented with YE were used for comparisons. Results are expressed as mean ± SD. Means for each bar without a common letter differ significantly. Significance with Tukey’s HSD *post hoc* test following a one-way ANOVA is indicated as *p* < 0.05.

In addition, the GABA-enriched FSJ was able to induce a significant reduction of intestinal and serum TNF-α, and to increase IFN-γ and IL-10 when compared to LPS-challenged controls (Figure 6). The treatments with *L. brevis* CRL 2013 and diluted GABA-enriched FSJ were also capable of increasing and reducing the levels of IL-10 and TNF-α, respectively. However, the concentrations of both cytokines in those groups did not reach the values found in the non-diluted FSJ group (Figure 6). On the other hand, the treatment of mice with the CRL 2013 strain induced a similar increase of intestinal and serum IFN-γ than the observed in the GABA-enriched FSJ (Figure 6). Animals treated with NFSJ or NFSJ supplemented with YE had values of peritoneal (Figure 5), intestinal and serum cytokines (Figure 6) that were not different from control mice in the context of TLR4-induced inflammation (Figure 5).

**FIGURE 7:**
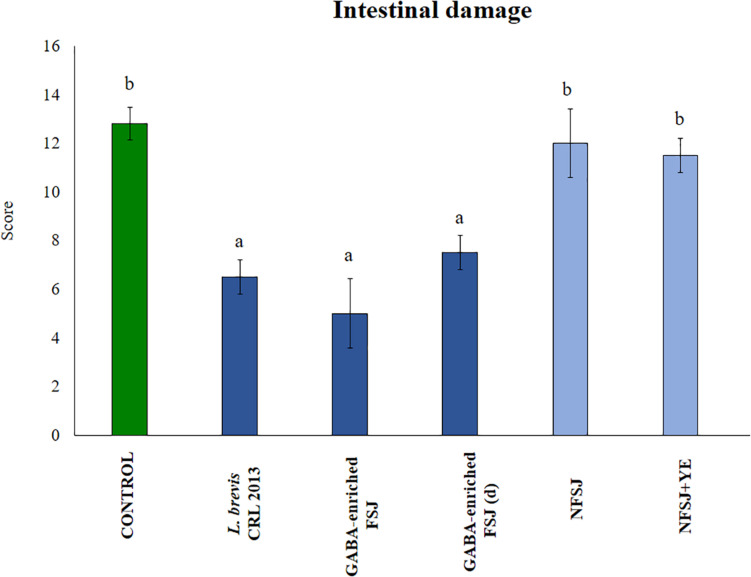
Effect of *L. brevis* CRL 2013, GABA-enriched strawberry fermented juice (FSJ) and diluted (d) GABA-enriched FSJ on intestinal damage in mice challenged with LPS. Animals treated with NFSJ or NFSJ supplemented with YE were used for comparisons. Score values were calculated considering the presence/absence and intensity of edema, epithelial injury, degranulation of Paneth cells and inflammation. Results are expressed as mean ± SD. Means for each bar without a common letter differ significantly. Significance with Tukey’s HSD *post hoc* test following a one-way ANOVA is indicated as *p* < 0.05.

The histopathological examination of the intestinal tissue revealed that LPS administration was capable of increasing the damage score from 4 (normal score) up to ∼14 (Figure 7). Indeed, the LPS challenge induced intestinal edema, epithelial injury, infiltration of inflammatory cells and a moderate degranulation of Paneth cells. The treatments with non-diluted and diluted GABA-enriched FSJ as well as with *L. brevis* CRL 2013 significantly reduced the scores of intestinal injury. Of note, although there were not statistical significant differences between the score of these three groups, mice receiving the non-diluted GABA-enriched FSJ had a tendency to lower infiltration of inflammatory cells in the intestinal mucosa. Animals treated with NFSJ or NFSJ supplemented with YE had values of intestinal injury scores that were not different from control mice (Figure 7).

## Discussion

Gamma-aminobutyric acid is a non-protein amino acid that has been extensively described as a health-promoting functional compound (Diana et al., 2014; Quílez and Diana, 2017). Considering that berries contain powerful antioxidants and other functional compounds, biotechnological approaches are being currently used to increase the content of specific health-related compounds in these fruits and its derivatives (Battino et al., 2009). Since LAB are currently the most interesting group of microorganisms capable of producing GABA at high yields, there is an opportunity to isolate and identify GABA producing strains to be used as starter cultures in the design and development of novel functional fermented foods or as probiotics. In this regard, based on previous screenings performed in our laboratory (Cataldo et al., 2020), *L. brevis* CRL 2013 was selected as the most appropriate GABA-producing LAB for the manufacture of a functional fermented beverage.

Since lactic acid fermentation has a remarkable role in improving the intrinsic functional and nutritional properties of many vegetable food matrixes (Di Cagno et al., 2016) and due to its well-known positive effects in traditional fermented foods, in this work both blueberry and strawberry juices were subjected to a process of lactic fermentation by *L. brevis* CRL 2013. The acidification rate during both strawberry and blueberry fermentation was similar to that observed by Kim et al. (2009) when fermenting a black raspberry juice with *L. brevis* GABA 100. In order to set the vegetable matrix for the fermentation, additional sources of nitrogen were added to the juices, an alternative also used by Di Cagno et al. (2010) when fermenting a grape must. A survey of the genome of CRL 2013 strain (Accession number: NZ_MZMW00000000.1) confirms that *L. brevis* CRL 2013 lacks the gene encoding cell envelope associated proteinase. Since *L. brevis* CRL 2013 is a non-proteolytic strain, the growth recovery was probably due to the presence of several free amino acids and peptides in both YE and tryptein, which are essential for bacterial growth (Hebert et al., 2001). Although GABA synthesis began earlier in the strawberry juice, it continued to progress up to 168 h in both juices, reaching a maximum GABA level of 262 mM GABA. In this sense, GABA in raspberry juices fermented by *L. brevis* GABA 100 at 30°C reached the maximum levels on the 12th day (Kim et al., 2009). To our knowledge, this is the first report regarding the GABA-enrichment of blueberry and strawberry juices by lactic acid fermentation.

The functional *in vitro* and *in vivo* studies performed in this work allow us to reach three important conclusions: (a) the GABA-enriched FSJ produced with *L. brevis* CRL 2013 is able to differentially modulate the inflammatory response induced by the activation of TLR4; (b) the immunoregulatory effect induced by the GABA-enriched FSJ depends on its concentration, and (c) the intrinsic immunomodulatory properties of the CRL 2013 strain may contribute to the modulatory effect of the GABA-enriched FSJ.

In our hands, the GABA-enriched FSJ was capable of reducing the expression of the inflammatory marker *cox-2* in RAW macrophages activated by TLR4 stimulation. In line with our results, it was reported previously that GABA is capable of inhibiting immune cells activation by modulating NF-kB pathway, thus diminishing the production of inflammatory mediators such as TNF-α, COX-2 and iNOS (Jin et al., 2013; Prud’homme et al., 2015). In addition, the ability of GABA to inhibit the *in vitro* release of IL-6 and IL-12 by LPS-stimulated peritoneal macrophages was also reported by Reyes-García et al. (2007). Besides, it was shown that a GABA-enriched pepino extract obtained after fermentation by *L. brevis* BCRC 12310 was able to inhibit the expression of TNF-α in LPS-induced RAW 264.7 macrophages (Chang et al., 2016).

**FIGURE 8:**
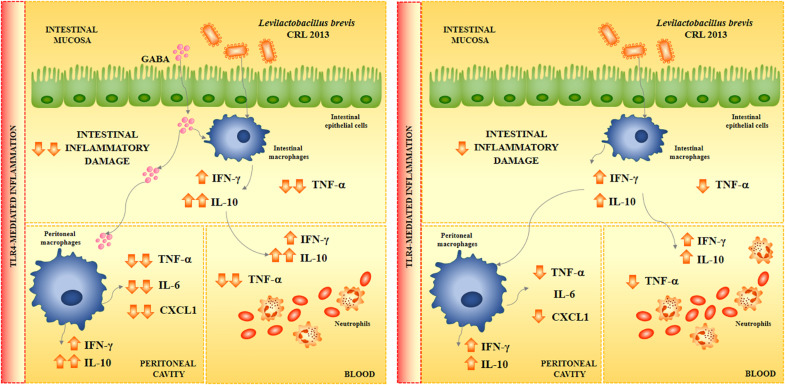
Proposed mechanism for the immunomodulatory effect of *Levilactobacillus brevis* CRL 2013 and GABA in the immune response triggered by the activation of TLR4.

To the best of our knowledge, the immunoregulatory effects of GABA-enriched products after fermentation with lactobacilli have not been studied in depth within *in vivo* models. For this reason, we also aimed in this work to demonstrate *in vivo* the potential immunomodulatory effect of the GABA-enriched FSJ obtained after the fermentation with the CRL 2013 strain. We were able to demonstrate that the oral treatment with the GABA-enriched FSJ was capable of modulating the inflammatory response triggered by the activation of TRL4 at the intestinal mucosa, in the peritoneal cavity and at the systemic level. The GABA-enriched FSJ reduced the production of TNF-α, IL-6 and CXCL1 as well as improved the levels of IL-10 in the three body compartments analyzed (Figure 8). Activation of TLR in macrophages after the binding of its ligand induce a signaling cascade that culminates in the expression and secretion of various cytokines, chemokines, and other inflammatory factors which signal and prime neighboring immune cells. Although necessary to confer protection against invading pathogens, the TLR-mediated inflammation needs to be tightly controlled at mucosal surfaces such as the intestinal tissue to avoid exaggerated inflammatory responses and damage. Then, we speculated that the differential cytokine profile induced by the GABA-enriched FSJ in the intestinal mucosa after TLR4 activation would be capable of protecting against the inflammatory damage. The histological studies performed here, demonstrated that this statement was correct since a significantly lower intestinal damage score, mainly related to a lower infiltration of inflammatory cells, was observed in mice treated with the GABA-enriched FSJ when compared to controls.

Interestingly, it has been shown that GABA-enriched products obtained by fermentation with lactobacilli can modulate not only the immune response mediated by macrophages but also by other immune cells such as lymphocytes. In this regard, it was recently reported that live bacteria-free supernatant collected from the GABA-producing *L. brevis* BGZLS10-17 is able to differentially modulate the proliferation, the MHCII and CD80 expression and the production of IFN-γ and IL-17 in concanavalin A-stimulated mesenteric lymph node lymphocytes (Bajić et al., 2020). Furthermore, GABA-containing supernatants were able to increase the expression of immunoregulatory molecules Foxp3, IL-10, and TGF-β in immune cells. These *in vitro* findings correlated with the *in vivo* ability of the GABA producing BGZLS10-17 strain to protect against inflammatory-induced destruction of intestinal barrier in an experimental autoimmune encephalomyelitis model (Sokovic Bajic et al., 2019). These results indicate that it would be of great importance to study the immunomodulatory effect of the CRL 2013-derived GABA-enriched FSJ in the context of other inflammatory challenges, to know its potential to be applied in the prevention or treatment of autoimmune and inflammatory diseases.

In the present study, we also observed a correlation between GABA concentration and the degree of repression of *cox-2* in murine macrophages activated by TLR4 stimulation; 1 mM GABA pretreatment presented inhibitory influence whereas 0.1 mM GABA was not enough concentration to achieve a significant inhibition. This dose-dependent inhibition is in agreement with a previous result in which 1 mM GABA was the optimal concentration to reach the highest inhibition on the expression of TNF-α and iNOS in LPS-induced RAW 264.7 cells (Han et al., 2007). Furthermore, these *in vitro* results are in line with the *in vivo* studies performed here that demonstrated the reduced ability of the diluted GABA-enriched FSJ to modulate the inflammatory response triggered by TLR4 activation in mice when compared to non-diluted GABA-enriched FSJ. It should be noted that another possibility for the lower ability of the diluted GABA-enriched FSJ to modulate the innate immune response could be related to the dilution of the lactobacilli dose. It would be interesting to study in the future the effect of different doses of *L. brevis* CRL 2013 and concentrations of GABA-enriched FSJ to modulate the TLR4-triggered inflammation to find the optimal doses that exert their beneficial effects.

Interestingly, the oral administration of *L. brevis* CRL 2013 to mice was able to increase the intestinal, peritoneal and serum levels of IFN-γ and IL-10. Several studies have reported the beneficial effects of immunomodulatory lactobacilli and have highlighted that the most remarkable effect of lactobacilli on the intestinal cytokine profile is the increase of TNF-α, IFN-γ, and IL-10 (Salva et al., 2010; Marranzino et al., 2012; Villena et al., 2012). Moreover, it is considered that through the release of intestinal TNF-α and IFN-γ, immune enhancing lactobacilli are capable of stimulating the activities of peritoneal macrophages. In this regard, we have shown that the oral administration of the highly immune enhancing strains *Lacticaseibacillus casei* CRL431 (formerly known as *Lactobacillus casei* CRL431), *Lactiplantibacillus plantarum* CRL1506 (formerly known as *Lactobacillus plantarum* CRL1506), and *Lacticaseibacillus rhamnosus* CRL1505 (formerly known as *Lactobacillus rhamnosus* CRL1505) increase the production of TNF-α, IFN-γ, and IL-10 in the intestine after their oral administration (Salva et al., 2010; Marranzino et al., 2012; Villena et al., 2012). More recently, we observed that immunomodulatory strains such as *Limosilactobacillus fermentum* UCO-979C (formerly known as *Lactobacillu fermentum* UCO-979C) significantly improved the production of intestinal IFN-γ, and IL-10 but not of TNF-α (Garcia-Castillo et al., 2019). This probiotic strain is not a highly stimulating strain of the immune system, but rather behaves like an immunoregulatory strain, moderately stimulating effector responses and inducing anti-inflammatory responses. The results obtained in this work, allow concluding that the CRL 2013 strain belongs to this group of lactic acid bacteria. In fact, *L. brevis* CRL 2013-treated mice were able to differentially regulate the response to TLR4 by reducing the production of TNF-α and CXCL1, and further enhancing IL-10 (Figure 8).

Some studies have described immune enhancing *L. brevis* strains. The treatment of macrophages with *L. brevis* KCTC 12777BP increased their phagocytic activity and the production of TNF-α, IL-6, and nitric oxide (Jeong et al., 2020). *L. brevis* ZLB004 was capable of increasing IFN-γ concentration enhancing the immune health status of weaned pigs (Liu et al., 2015) while *L. brevis* ATCC 8287 reduced TGF-β1 and increased IL-6 expressions in the small intestine of pigs (Lähteinen et al., 2014). However, no strains of *L. brevis* have been described to possess a mixed stimulatory/anti-inflammatory profile. Then, our results raise the question of whether *L. brevis* CRL 2013 could be used by itself as an immunomodulatory probiotic strain to exert beneficial effects in other immune-related diseases. It should be noted that the intrinsic anti-inflammatory activity of the CRL 2013 strain was increased when it was administered together with GABA. However, it is possible to rule out the effect of GABA in the bacteria administered alone, since the concentrations of GABA that this bacterium can produce *in situ* in the intestinal mucosa are far below those necessary to obtain the immunomodulatory effect, as demonstrated by our comparative studies using non-diluted and diluted GABA-enriched FSJ. Then, it would also be of great value to investigate which bacterial molecule(s) are responsible for the intrinsic immunomodulatory effect observed in the CRL 2013 strain.

In conclusion, *L. brevis* CRL 2013, a major GABA producer among the strains evaluated in our laboratory, was used for the formulation of a GABA-enriched fermented berry juice. Considering that a significantly higher GABA yield was observed in fermented strawberry than in fermented blueberry juice, the former one was selected for further studies. The highest GABA production was obtained by fermenting the YE-supplemented strawberry juice with *L. brevis* CRL 2013 as starter culture. The GABA-enriched strawberry juice modulated the expression of *cox-2* in LPS stimulated RAW 264.7 macrophages and exerted a remarkable anti-inflammatory effect *in vivo* in the context of TLR4 activation. Our observations strikingly support the potential of GABA and GABA-enriched strawberry fermented juices as promising functional foods to help ameliorate the exacerbated inflammatory response of chronic inflammatory diseases in addition to the other well-known GABA positive properties such as diuretic, antihypertensive, hypoglycemic and antidepressant compound. To our knowledge, this is the first report of a bio-enriched fermented strawberry juice capable of positively modulating the TLR4-mediated inflammatory response.

## Data Availability Statement

The raw data supporting the conclusions of this article will be made available by the authors, without undue reservation.

## Ethics Statement

The animal study was reviewed and approved by The CERELA-CONICET Institutional Animal Care and Use Committee prospectively approved this research under the protocol BIOT-CRL-17.

## Author Contributions

PC conducted most of the experiments, analyzed the results, and wrote the manuscript. EH conceived the idea for the project. LS coordinated and contributed in cell culture and RT-PCR assays. JV, LS, and EH coordinated the study and wrote the manuscript. GS and ME contributed to the discussion of the manuscript. All authors read and approved the final manuscript.

## Conflict of Interest

The authors declare that the research was conducted in the absence of any commercial or financial relationships that could be construed as a potential conflict of interest.

## References

[B1] Abdou A. M. Higashiguchi S. Horie K. Kim M. Hatta H. Yokogoshi H. (2006). Relaxation and immunity enhancement effects of gamma-aminobutyric acid (GABA) administration in humans. *Biofactors* 26 201–208. 10.1002/biof.5520260305 16971751

[B2] Adeghate E. Ponery A. S. (2002). GABA in the endocrine pancreas: cellular localization and function in normal and diabetic rats. *Tissue Cell* 34 1–6. 10.1054/tice.2002.0217 11989965

[B3] Bajić S. S. Ðokić J. Dinić M. Tomić S. Popović N. Brdarić E. (2020). GABA potentiate the immunoregulatory effects of *Lactobacillus brevis* BGZLS10-17 via ATG5-dependent autophagy *in vitro*. *Sci. Rep.* 10:1347. 10.1038/s41598-020-58177-2 31992761 PMC6987229

[B4] Bao W. Huang X. Liu J. Han B. Chen J. (2020). Influence of *Lactobacillus brevis* on metabolite changes in bacteria-fermented sufu. *J. Food Sci.* 85 165–172. 10.1111/1750-3841.14968 31898817

[B5] Battino M. Beekwilder J. Denoyes-Rothan B. Laimer M. Mcdougall G. J. Mezzetti B. (2009). Bioactive compounds in berries relevant to human health. *Nutr. Rev.* 67(Suppl. 1), S145–S150. 10.1111/j.1753-4887.2009.00178.x 19453670

[B6] Brown L. Villegas J. M. Elean M. Fadda S. Mozzi F. Saavedra L. (2017). YebC, a putative transcriptional factor involved in the regulation of the proteolytic system of *Lactobacillus*. *Sci. Rep.* 7:8579. 10.1038/s41598-017-09124-1 28819300 PMC5561223

[B7] Cataldo P. G. Villegas J. M. Savoy De Giori G. Saavedra L. Hebert E. M. (2020). Enhancement of γ-aminobutyric acid (GABA) production by *Lactobacillus brevis* CRL 2013 based on carbohydrate fermentation. *Int. J. Food Microbiol.* 333:108792. 10.1016/j.ijfoodmicro.2020.108792 32707524

[B8] Chang V. H. Chiu T. H. Fu S. C. (2016). *In vitro* anti-inflammatory properties of fermented pepino (*Solanum muricatum*) milk by gamma-aminobutyric acid-producing *Lactobacillus brevis* and an *in vivo* animal model for evaluating its effects on hypertension. *J. Sci. Food Agric.* 96 192–198. 10.1002/jsfa.7081 25582456

[B9] Cui Y. Miao K. Niyaphorn S. Qu X. (2020). Production of gamma-aminobutyric acid from lactic acid bacteria: a systematic review. *Int. J. Mol. Sci.* 21:995. 10.3390/ijms21030995 32028587 PMC7037312

[B10] Di Cagno R. Filannino P. Gobbetti M. (2016). “Novel fermented fruit and vegetable-based products,” in *Novel Food Fermentation Technologies*, eds Oija K. S. Tiwari B. K. (Cham: Springer International Publishing), 279–291. 10.1007/978-3-319-42457-6_13

[B11] Di Cagno R. Mazzacane F. Rizzello C. G. De Angelis M. Giuliani G. Meloni M. (2010). Synthesis of gamma-aminobutyric acid (GABA) by *Lactobacillus plantarum* DSM19463: functional grape must beverage and dermatological applications. *Appl. Microbiol. Biotechnol.* 86 731–741. 10.1007/s00253-009-2370-4 20013120

[B12] Diana M. Quílez J. Rafecas M. (2014). Gamma-aminobutyric acid as a bioactive compound in foods: a review. *J. Funct. Foods* 10 407–420. 10.1016/j.jff.2014.07.004

[B13] Efsa Panel on Dietetic Products Nutrition, and Allergies [Nda]. (2009). Scientific Opinion on the substantiation of health claims related to gamma-aminobutyric acid and cognitive function (ID 1768) pursuant to article 13(1) of regulation (EC) No 1924/2006. *EFSA J.* 7:1274. 10.2903/j.efsa.2009.1274 29606757

[B14] Elean M. Albarracin L. Cataldo P. G. Londero A. Kitazawa H. Saavedra L. (2020). New immunobiotics from highly proteolytic *Lactobacillus delbrueckii* strains: their impact on intestinal antiviral innate immune response. *Benef. Microbes* 11 375–390. 10.3920/BM2019.0198 32755264

[B15] Garcia-Castillo V. Komatsu R. Clua P. Indo Y. Takagi M. Salva S. (2019). Evaluation of the immunomodulatory activities of the probiotic strain *Lactobacillus fermentum* UCO-979C. *Front. Immunol.* 10:1376. 10.3389/fimmu.2019.01376 31263467 PMC6585165

[B16] Han D. Kim H. Y. Lee H. J. Shim I. Hahm D. H. (2007). Wound healing activity of gamma-aminobutyric Acid (GABA) in rats. *J. Microbiol. Biotechnol.* 17 1661–1669. 18156782

[B17] Hebert E. M. De Giori G. S. Raya R. R. (2001). Isolation and characterization of a slowly milk-coagulating variant of *Lactobacillus helveticus* deficient in purine biosynthesis. *Appl. Environ. Microbiol.* 67 1846–1850. 10.1128/AEM.67.4.1846-1850.2001 11282642 PMC92806

[B18] Jeong M. Kim J. H. Lee J. S. Kang S. D. Shim S. Jung M. Y. (2020). Heat-killed *Lactobacillus brevis* enhances phagocytic activity and generates immune-stimulatory effects through activating the TAK1 pathway. *J. Microbiol. Biotechnol.* 30 1395–1403. 10.4014/jmb.2002.02004 32627755 PMC9728231

[B19] Jin Z. Mendu S. K. Birnir B. (2013). GABA is an effective immunomodulatory molecule. *Amino Acids* 45 87–94. 10.1007/s00726-011-1193-7 22160261 PMC3680704

[B20] Johansen E. (2017). Future access and improvement of industrial lactic acid bacteria cultures. *Microb. Cell Fact.* 16:230. 10.1186/s12934-017-0851-1 29268733 PMC5738899

[B21] Kim J. Y. Lee M. Y. Ji G. E. Lee Y. S. Hwang K. T. (2009). Production of gamma-aminobutyric acid in black raspberry juice during fermentation by *Lactobacillus brevis* GABA100. *Int. J. Food Microbiol.* 130 12–16. 10.1016/j.ijfoodmicro.2008.12.028 19167126

[B22] Lähteinen T. Lindholm A. Rinttilä T. Junnikkala S. Kant R. Pietilä T. E. (2014). Effect of *Lactobacillus brevis* ATCC 8287 as a feeding supplement on the performance and immune function of piglets. *Vet. Immunol. Immunopathol.* 158 14–25. 10.1016/j.vetimm.2013.09.002 24074625

[B23] Lee A. K. Sung S. H. Kim Y. C. Kim S. G. (2003). Inhibition of lipopolysaccharide-inducible nitric oxide synthase, TNF-alpha and COX-2 expression by sauchinone effects on I-kappaBalpha phosphorylation, C/EBP and AP-1 activation. *Br. J. Pharmacol.* 139 11–20. 10.1038/sj.bjp.0705231 12746218 PMC1573829

[B24] Li W. Wei M. Wu J. Rui X. Dong M. (2016). Novel fermented chickpea milk with enhanced level of gamma-aminobutyric acid and neuroprotective effect on PC12 cells. *PeerJ* 4:e2292. 10.7717/peerj.2292 27602272 PMC4991855

[B25] Liu H. Ji H. F. Zhang D. Y. Wang S. X. Wang J. Shan D. C. (2015). Effects of *Lactobacillus brevis* preparation on growth performance, fecal microflora and serum profile in weaned pigs. *Livest. Sci.* 178 251–254. 10.1016/j.livsci.2015.06.002

[B26] Livak K. J. Schmittgen T. D. (2001). Analysis of relative gene expression data using real-time quantitative PCR and the 2(-Delta Delta C(T)) method. *Methods* 25 402–408. 10.1006/meth.2001.1262 11846609

[B27] Marcial G. Sendker J. Brandt S. De Lampasona M. P. Catalan C. A. De Valdez G. F. (2014). Gastroprotection as an example: antiadhesion against *Helicobacter pylori*, anti-inflammatory and antioxidant activities of aqueous extracts from the aerial parts of *Lippia integrifolia Hieron*. *J. Ethnopharmacol.* 155 1125–1133. 10.1016/j.jep.2014.06.039 24993887

[B28] Marranzino G. Villena J. Salva S. Alvarez S. (2012). Stimulation of macrophages by immunobiotic *Lactobacillus* strains: influence beyond the intestinal tract. *Microbiol. Immunol.* 56 771–781. 10.1111/j.1348-0421.2012.00495.x 22846065

[B29] Nile S. H. Park S. W. (2014). Edible berries: bioactive components and their effect on human health. *Nutrition* 30 134–144. 10.1016/j.nut.2013.04.007 24012283

[B30] Park K. B. Oh S. H. (2007). Production of yogurt with enhanced levels of gamma-aminobutyric acid and valuable nutrients using lactic acid bacteria and germinated soybean extract. *Bioresour. Technol.* 98 1675–1679. 10.1016/j.biortech.2006.06.006 17055264

[B31] Prud’homme G. J. Glinka Y. Wang Q. (2015). Immunological GABAergic interactions and therapeutic applications in autoimmune diseases. *Autoimmun. Rev.* 14 1048–1056. 10.1016/j.autrev.2015.07.011 26226414

[B32] Quílez J. Diana M. (2017). “Chapter 5 - gamma-aminobutyric acid-enriched fermented foods,” in *Fermented Foods in Health and Disease Prevention*, eds Frias J. Martinez-Villaluenga C. Peñas E. (Boston: Academic Press), 85–103. 10.1016/b978-0-12-802309-9.00005-4

[B33] Ramos-Ruiz R. Poirot E. Flores-Mosquera M. (2018). GABA, a non-protein amino acid ubiquitous in food matrices. *Cogent. Food Agric.* 4:1534323 10.1080/23311932.2018.1534323

[B34] Reyes-García M. G. Hernández-Hernández F. Hernández-Téllez B. García-Tamayo F. (2007). GABA (A) receptor subunits RNA expression in mice peritoneal macrophages modulate their IL-6/IL-12 production. *J. Neuroimmunol.* 188 64–68. 10.1016/j.jneuroim.2007.05.013 17599468

[B35] Salva S. Villena J. Alvarez S. (2010). Immunomodulatory activity of *Lactobacillus rhamnosus* strains isolated from goat milk: impact on intestinal and respiratory infections. *Int. J. Food Microbiol.* 141 82–89. 10.1016/j.ijfoodmicro.2010.03.013 20395002

[B36] Sokovic Bajic S. Djokic J. Dinic M. Veljovic K. Golic N. Mihajlovic S. (2019). GABA-Producing natural dairy isolate from artisanal Zlatar cheese attenuates gut inflammation and strengthens gut epithelial barrier *in vitro*. *Front. Microbiol.* 10:527. 10.3389/fmicb.2019.00527 30936860 PMC6431637

[B37] Taranukhin A. G. Saransaari P. Kiianmaa K. Gunnar T. Oja S. S. (2017). Comparison of toxicity of taurine and GABA in combination with alcohol in 7-day-old mice. *Adv. Exp. Med. Biol.* 975(Pt 2), 1021–1033. 10.1007/978-94-024-1079-2_81 28849519

[B38] Torres M. J. Villena J. Barroso P. Fanciotti M. N. Audisio M. (2017). Safety Assessment of the antimicrobial lipopeptides synthesized by *Bacillus subtilis* subsp. subtilis CBMDC3f. EC. *Microbiology* 8.3 113–122.

[B39] Tsukatani T. Higuchi T. Matsumoto K. (2005). Enzyme-based microtiter plate assay for γ-aminobutyric acid: application to the screening of γ-aminobutyric acid producing lactic acid bacteria. *Anal. Chim. Acta* 540 293–297. 10.1016/j.aca.2005.03.056

[B40] Villegas J. M. Brown L. Savoy De Giori G. Hebert E. M. (2016). Optimization of batch culture conditions for GABA production by *Lactobacillus brevis* CRL 1942, isolated from quinoa sourdough. *LWT Food Sci. Technol.* 67 22–26. 10.1016/j.lwt.2015.11.027

[B41] Villena J. Chiba E. Tomosada Y. Salva S. Marranzino G. Kitazawa H. (2012). Orally administered *Lactobacillus rhamnosus* modulates the respiratory immune response triggered by the viral pathogen-associated molecular pattern poly(I:C). *BMC Immunol.* 13:53. 10.1186/1471-2172-13-53 22989047 PMC3460727

[B42] Wu Q. Law Y. S. Shah N. P. (2015). Dairy Streptococcus thermophilus improves cell viability of *Lactobacillus brevis* NPS-QW-145 and its gamma-aminobutyric acid biosynthesis ability in milk. *Sci. Rep.* 5:12885. 10.1038/srep12885 26245488 PMC4526857

[B43] Yang N.-C. Jhou K.-Y. Tseng C. Y. (2012). Antihypertensive effect of mulberry leaf aqueous extract containing γ-aminobutyric acid in spontaneously hypertensive rats. *Food Chem.* 132 1796–1801. 10.1016/j.foodchem.2011.11.143

