# Supplementary figures and images for: Immunomodulatory Properties of a γ-Aminobutyric Acid-Enriched Strawberry Juice Produced by Levilactobacillus brevis CRL 2013
Source: Front Microbiol. 2020 Dec 17;11:610016. doi: 10.3389/fmicb.2020.610016 (PMC7773669; doi:10.3389/fmicb.2020.610016)

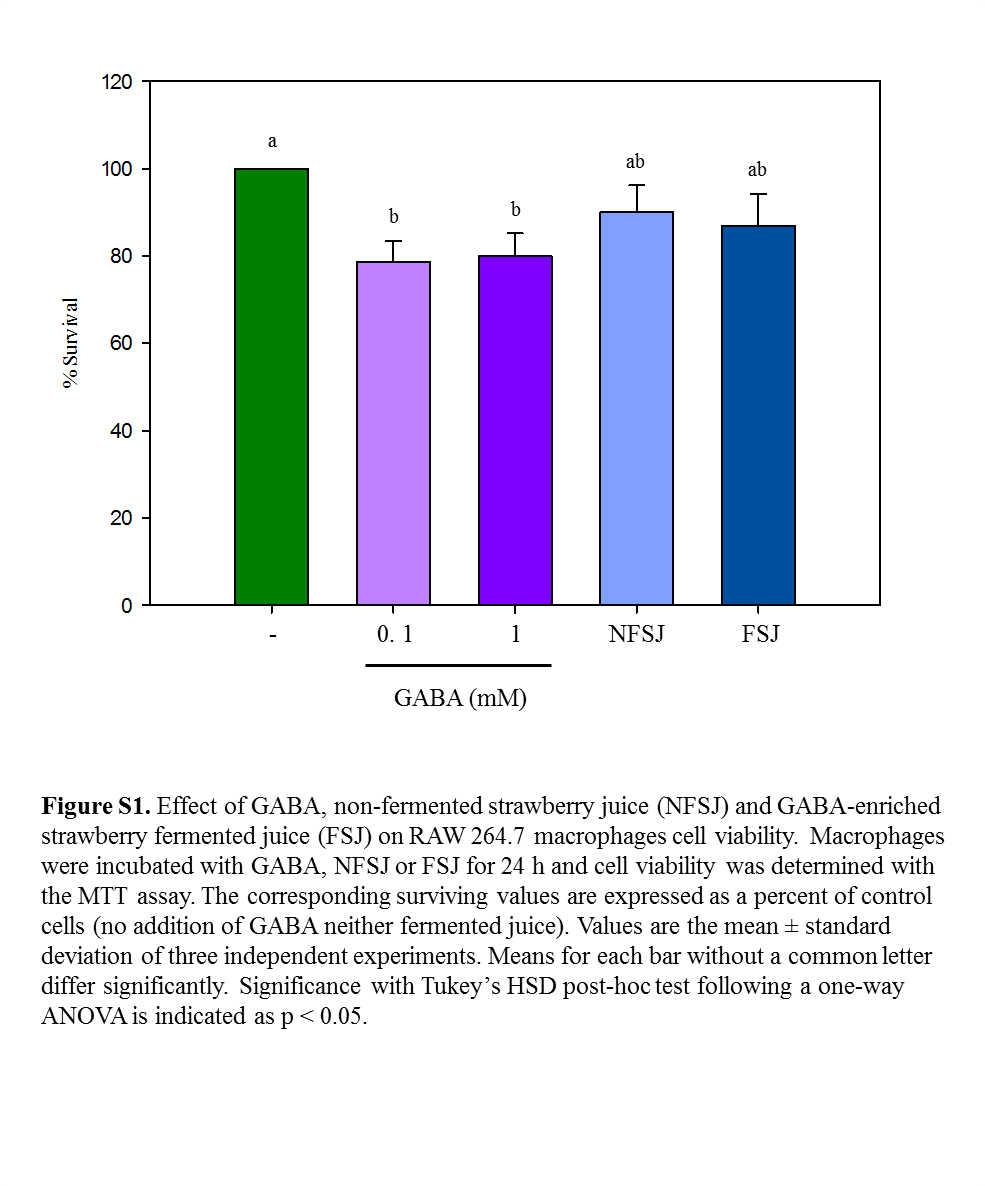

Supplement: Supplementary file 1 [file Image_1.tif]
